# Plant-derived saponins and their prospective for cosmetic and personal care products

**DOI:** 10.1186/s40529-024-00438-8

**Published:** 2024-11-08

**Authors:** Mayuree Kanlayavattanakul, Donia Mersni, Nattaya Lourith

**Affiliations:** 1 School of Cosmetic Science, Mae Fah Luang University, Chiang Rai, 57100 Thailand; 2 Phytocosmetics and Cosmeceuticals Research Group, Mae Fah Luang University, Chiang Rai, 57100 Thailand; 3 Nantes-Atlantic National College of Veterinary Medicine, Food Science and Engineering, Rue de la Geraudiere, CS 82225, Nantes, 44322 France

**Keywords:** Saponin, Plants, Cosmetics, Bio-based materials, Industrial crops

## Abstract

Plants are industrially cultivated and processed serving for specified sectors for human consumptions including cosmetic and personal care products. Where, the consumers’ awareness towards sustainability are increasing year by year. Among which, those of the materials derived from the plants produced with good agricultural and manufacturing practices abided with bio-circular-green economy theme, are of eminence. This perspective is in line with the researchers’ bioprospective onto natural products. Special attention sheds on saponins, the biosurfactants that will not cause detrimental effects on the environment. Which, plants are regarded as the sustainable sources of these cosmetic substances. However, among tremendous plants that have been continuously explored upon their potential applications. Most of the studies focus on preparation of the saponins and biological activities. Surprisingly, those that are abided with the list published in the European Commission (CosIng) that are of crucially for cosmetic regulation are insufficiently demonstrated, which burden their applications in the sector. This context summarizes the industrial crops that are registered as plant saponin in the CosIng database. Those that are insufficiently exploited on the information required for cosmetic formulations are therefore encouraged to be examined. In addition, multidirectional cosmetic beneficials of the filled plants saponin would be encouraged to be explored. These plants will be properly knowledge managed for their sustainable utilizations as the bio-based materials promising for cosmetic and personal care industrial perspectives.

## Introduction

Plants have been shown to be the sustainable sources for functional and specialty materials applicable in variety of the products commercializing in the market (Rai et al. 2021). Cosmetic and personal care sectors are the major industries where natural and sustainable demands are increasing, which the industrial crops are regarded as the reliable sources suppling the demands for these materials (Bravo et al. 2020) including those of surface-active ones that are vastly applied in household detergent and cleansing products (Rai et al. 2021) particularly saponins (Jolly et al. 2023). In which, the natural materials are emerging among the consumers’ preferences towards sustainability and the conceivable more safety awareness, with multidirectional activity of the industrial crop-derived cosmetic substances (Chaikul et al. 2017; Lourith et al. 2016). Thus, the agricultural crops producing saponins are of significance to be explored in an order to achieve their applicability for high value products serving for the certain industries, i.e., fast moving consumer goods (FMCGs) that the consumers’ expectation among the natural derived product sustainably implied from the orient crops is emerging. In addition, the plants’ applicability choices would be expanded in regarded with their multidirectional activities. Agricultural sustainability management for the industrial crops production would be the key answer fitting with the emerging challenge, i.e., circular bioeconomy, which cosmetics industry is addressing on this issue at all levels (Martins and Marto 2023).

### Plant saponins; the natural surface-active materials

Plants have long been served for human consumptions in a variety of products including cosmetics. Where, the plants applications are noted for positive effects for human as documented in several traditional medicines such as Ayurvedic, Chinese, Indusynunic, Islamic or Unani-Tibb, Kampo and Oriental recipes. Which, the traditional medicines that relied on plants that also modernly known as Herbalism or Homeopathy. Nowadays, majority applications of plants in cosmetics are popular world-wide and recognized in the term of “phytocosmetics”. Phytocosmetics is therefore refers to cosmetic preparation in any dosage form composing with plant i.e., plant extract (total extract or crude), selected active principles or isolated pure compounds from plants. At which the application of plant-derived ingredients in cosmetics will be by their functional and active tasks. Functional task is claimed on the basis of their functionalities in the formulation technology such as film-former, gelling agent, thickener, suspending agent, conditioner and emulsifier. That, mainly relies on their physicochemical properties. On the other hand, phytocosmetic actives are the efficacy tasks positively effects onto the aesthetic conditions of skin and hair i.e., anti-aging, skin lightening, skin hydrating/moisturizing, anti-greasiness, anti-dandruff, anti-microbial, hair color covering, anti-hair loss and so forth. The consumers’ awareness, acceptance and demand upon natural cosmetics are therefore clearly on phytocosmetics, where its’ expectation in the market is dramatically increasing year by year in regard on their perceivable safety and efficacy surplus with the consumers’ consideration on eco-friendly and sustainable products enlightening their importance in aesthetic benefits (Lourith and Kanlayavattanakul 2021).

Surface-materials so called surfactants/emulsifiers are applied in cosmetic and personal care products in regard with their functional properties, i.e., detergency, wetting, emulsifying, solubilizing, dispersing and foaming effects. These amphiphilic compounds containing hydrophobic and hydrophilic moieties are able to reduce surface tension and facilitate the formation of emulsions between liquids of different polarities. Which, almost half of all surfactants produced are for the washing and cleaning sectors. The interests toward the biosurfactants are dramatically increasing in regard with their mildness onto the sensitive skin users as per less detriment toward the environment (Kanlayavattanakul and Lourith 2010; Lourith and Kanlayavattanakul 2009). Where, the plants-derived ones are of particular in focus in regards with their dermocosmetic beneficials in addition to their majorly functions, surface-active properties (Kanlayavattanakul and Lourith 2022). Which, saponins are the natural surface-active materials that are biosynthesized in plants as the industrial crops.

Saponins are secondary metabolites that are mainly distributed in plants (Jolly et al. 2023), where they are synthesized as the plants’ self-defense tasks against plant pathogens. Which plants producing saponins are obviously named with their soapy lather when shaken in water (Rai et al. 2021), i.e., soapbark, soapnut, soaproot and soapwort. These secondary metabolites have been utilized in several sectors of consumable products like taste modifiers, surfactants and emulsifiers as well as for drugs and medicines. Saponins molecules are diverse depends on the producing plants. Which, the saponins’ structures are composed with hydrophobic aglycones conjugated with hydrophilic sugar moieties, i.e., glucuronic acid, galacturonic acid, rhamnose, arabinose, xylose and fructose. Accordingly, interfacial tension activity of the molecules is provided, and of crucially attributed by the reducing sugars of uronic acid homologues (Wojciechowski et al. 2014). Interfacial tension reduction mechanism of saponins is ruled by their high adsorption property be means of diffusion and barrier controlled as well. Which, uronic acid moieties of the hydrophilic part decrease electrostatic repulsion between saponin molecules and water phase achieving the adsorption and reduce surface tension consequently. Saponins are differentiated into triterpenoid, steroid and alkaloid saponins on the basis of the structures. Triterpenoid saponins are constructed with 6 units of a five-carbon isoprene, are exhibited as the most widely distributed saponins in plants. Moreover, they are better in surface activity than the steroid saponins (Golemanov et al. 2013). In addition, saponins are categorizable on the basis of the sugar homologues into mono-, di- and tridesmosidic saponins. In which, different parts of the same plant are varied in saponin structures elaborating different surface activity in turn. Which, a greater number of sugar moieties enhance a greater adsorption resulting in a superior interfacial tension activity accordingly. In term of sugar homologues, the greater molecular weight ones take a longer time on their molecular arrangement at the interface (Böttcher and Drusch 2016), which may govern by the steric hindrance of the sugar molecules, and slower reduce the surface tension accordingly. Furthermore, sugar moieties liberate different degree of skin hydrating potency as well (Kanlayavattanakul and Lourith 2022).

In accordance to the amphiphilic nature of saponins, the structures are self-assembled in an order to optimize the molecular interactions of the saponins with the phases in a tendency mode for interfacial tension reduction. Which, self-assembled structures of saponins are formable in micelles, liquid crystal, bilayers, vesicle and microemulsions (Hiemenz and Rajagopalan 1997). It should be noted that saponins’ capability to reduce surface tension is of importance for cosmetic products. In addition, the ability to stabilize foam highlights the potential of the saponins to be applied in cosmetics. Which, the quality assessment of the saponins is undertaken by means of chemical reaction to quantify the quantity of saponins (total saponins content) as well as surface-active properties as followings.

### Characterization of saponins

Saponins are characterized by their surface-active properties in addition to their quantification in term of saponins content. In which, the surface activity of saponins is of crucial to be characterized for cosmetic and personal care preparations, in addition to the chemical characterization. There are several methods applicable for their characterization.

#### Total saponins content

Saponins can be quantified by means of vanillin-sulfuric acid assay in regard with its feasibility. Triterpenoid saponins react with vanillin, which the distinctive reactant color is oxidized by sulfuric acid. Chemicals and reagents for this assay are diluted in alcoholic water, which an absorption of the reaction solvent is needed to be cutoff. Thereafter, the spectrophotometric quantification of the total saponins content can be calculated. Total saponins content is reported, which the preliminary data obtained shall optimistically design further characterization. However, there are additional acids that shall be utilized as oxidants such as perchloric acid. Accordingly, the reactant color is varied, and the working wavelength is differed in turn. In addition, there can be a difference in the standard used as per the concentration and reaction time and temperature. Which, may infeasible for a comparative discussion between different studies.

Steroidal saponins can be quantified by the spectrophotometric method as well. Which, anisaldehyde is reacted with the saponins instead of vanillin. It should be noted that the reagents are prepared in ethyl acetate. Thus, working wavelength is differed from the triterpenoid saponins assessment.

In addition to the saponins content that is UV-Vis spectrophotometric measurable by means of vanillin-sulfuric acid assay. Quantitative using HPLC is of common for the know saponins’ quantification (Oleszek and Bialy 2006). That might be determined with mass spectrophotometry or by means of diode array detectors. Furthermore, FTIR analysis of saponins commonly applied for the saponins’ structure on the basis of aglycone and sugar moieties.

#### Surface tension

The saponins solution of curtained concentration and volume is instrumentally surface tension measured using a drop shape tensiometer. The interfacial tension is acquired maintaining a drop of the solution of about 30 mm^3^ at constant area after its rapid creation at the tip of a hydrophobized stainless steel capillary. In addition, dilational viscoelasticity can be acquired with the drop area deformed with the applied oscillation frequencies (Gόral and Wojciechowski 2020; Santini et al. 2019). In addition to drop method, Wilhelmy plate is advisable for surface tension measurement. At which, different concentrations of the saponins solution were measured following a stirring for 30 s and 300 s equilibration time at 25 ºC (Ralla et al. 2017). Du Noüy ring method is one of the commonly technique examining surface tension. Which, the saponins solution is being examined at the Du Noüy ring moving speed of 2 mm/s with a 3.5 mm immersion depth with a total of 20 measurements for 1 min at 25 ºC (Lunkenheimer and Wantke 1981).

#### Critical micelle concentration (CMC)

Critical micelle concentration (CMC) is one of the important quality characters of surfactants that is defined as the concentration of surfactants above which micelles are formed. CMC is assessable by several techniques on the basis of spectroscopic, tensiometric and conductometric methods.

Iodine-micelle complexation is applicable for CMC determination. Iodine solution mixing with different volume of saponins of the fixed concentration, adjusted to 10 ml and further record the absorbance over the working wavelength range 200–500 nm. The complexation of iodine with non-ionic association micelles, is thereafter determined at an absorption wavelength generated that is 225 nm. Consequently, a linear relation between absorbances and concentrations is obtained. Where CMC is calculated accordingly. Alternatively, CMC is determined by an α-lipoic acid micellization method. A fixed concentration of α-lipoic acid is mixed with a stock saponins solution at different volume and adjusted to 20 ml before absorbency recorded at 331 nm. The saponins-encapsulated α-lipoic acid becomes more soluble in aqueous solution and therefore absorbs more light. Which, the graph between the examined absorbances and surfactant concentrations is plotted, and CMC is therefore determined (Mesgarzadeh et al. 2017).

CMC is applicable to be determined by tensiometric method with a Wilhelmy plate. Different concentrations of the saponin samples shall be analyzed. CMC was obtained by calculating the intersection between the flat curve above the steep curve below the break in the interfacial tension isotherm (Böttcher and Drusch 2016).

The charge nature of the saponins can be observed on the basis of conductivity. The saponin solution is determined at different concentration, and later the relation between conductivity and concentration is plotted. Which, the ionic solutions of saponins are generated by means of the ionization of saponins’ carboxylic acids in the hydrophilic sugar moieties. Accordingly, the dissociated carboxylic anions thereby being responsible for conductivity (Rai et al. 2023), yields a linear dependency between the conductivity and the concentration. In contrary to the ionic solutions that there is a breaking point at the CMC with the slope of the curve being flatter above the CMC and steeper below the CMC. In addition to the conductivity assessment, plotting the surface tension retrieved from the surface tension measured at different concentrations of saponin extracts, CMC is determined with the onset of constancy in surface tension in spite of change in concentration.

#### Emulsification index

Emulsification index is able to be determined by mixing the saponin extract solution equally with paraffin liquid (2 ml, each), vigorous shaking for 2 min. The resulting mixture is kept overnight, where the solution is separated into emulsified and aqueous layers. Consequently, the index (E-24) is calculated by the ratio of the height of emulsified layer to the total height (Basu et al. 2015).

#### Foamability

Foam ability of the saponin solution can be examined by purging air into the solution through a porous membrane. Which, sintered glass or fritted glass with a known porosity is commonly applied for the porous membrane. The foam generated is thereafter recorded on heights versus time indicating foam stability index as well (Gόral and Wojciechowski 2020; Santini et al. 2019). In addition, the foam morphology can be observed with an optical microscope by placing the foam drop between 2 glass slides with a spacer of 0.2 mm thickness. Bubble size can be acquired using ImageJ software.

In addition to the aforementioned characteristics, stability of saponins is of crucially to be considered for cosmetic and personal care applications. Which, stability under different storage conditions, i.e., pH, ionic strength and temperature including the characteristics of the saponins challenged with different shear rates, are recommended to be verified. In an order to ensure the quality of the candidate saponins for cosmetic and personal care industrial applications.

### Industrial crops for plant saponins production

Saponins are utilized as functional cosmetic substances (Lourith and Kanlayavattanakul 2021) in regard with their surface activity. These bio-based materials are extractable from several plants and elicit variety tasks for cosmetic and personal care products as previously summarized (Jolly et al. 2023). Soapbark, soapwort, soap berry and quinoa are the common crops rich in saponins, where their names are tied with the surface reduction capability that are traditionally applied as soap.

#### *Quillaja saponaria*

*Quillaja saponaria* or soapbark is the common source of natural saponins that are commercially available. Quillaja saponins are approved to be used for human consumption with an acceptable daily intake (ADI) assigned by the FAO/WHO expert committee on food additive at 0–1 mg/kg body wt, and classified as GRAS (generally recognized as safe) by FEMA (flavor and extract manufactures’ association) number 2973. Bark of the plant aged of 30–50 years-old is extracted to produce Quillaja saponins. Thanks to the advancement of technology, nowadays the whole branches of the tree are saponin extractable. Quillaja saponins are serving in several industrial products (Reichert et al. 2019) in regard with the surface activity that self-assembling into micelles, liquid crystals, bilayers, vesicles and microemulsions with their CMC of 0.14–0.77 g/L (Mitra and Dungan 1997; Wojciechowski et al. 2014) as per biological activities on the basis of their natural origins for example anti-viral property against *Rhesus rotavirus* (RRV), the most common diarrheal pathogen in children, by disrupting the cellular membrane with therapeutic index of 2.95 (Tam and Roner 2011). In addition, cholesterol lowering effect of Quillaja saponins was reported (Vinarova et al. 2015). Quillaja extract is applied in cosmetics with CAS and EC no. of 68990-67-0 and 273-620-4, respectively, for claimed functions as cleansing, surfactant-emulsifying and foaming (European Commission, *Quillaja saponaria*) on the basis of its amphiphilic saponins.

#### *Saponaria officinalis*

*Saponaria officinalis* or soapwort is regarded as an important plant saponins used as household detergent (Sparg et al. 2004). Soapwort amphiphilic saponins are applicable in cosmetic products as well with its surface tension reduction of 49.1 ± 0.3 mN/m on the basis of saponin content of 50.92 ± 2.40 mg/g d.m. (Gόral et al. 2021).

#### *Sapindus mukorossi*

*Sapindus mukorossi* (soapberry or soapnut) is the common commercializing plant saponin. The triterpenoid saponins are formulated into cleansing products in regard with the detergency property. The saponins extract of soapberry with the reduced surface tension of 35.30 mN/m, and its’ indicated critical micelle concentration (CMC) of 7.50 × 10^3^ g/cc as examined by means of surface tension method (Pradhan and Bhattacharyya 2017). Biological activities of soapberry saponins fascinated in cosmetics. i.e., anti-inflammatory and anti-microbial activities (Hu et al. 2018; Wei et al. 2021). It should be noted that recently it is widely used as for an extraction of phytoactive compounds namely surfactant mediated extraction (SME) (Bhattacharya et al. 2021; Su et al. 2019).

#### *Chenopodium quinoa*

*Chenopodium quinoa* or quinoa has been cultivating worldwide recently, and is highlighted as a high nutritional food with several secondary metabolites beneficial for health including saponins (85–90% w/w) (Ruiz et al. 2017) that are mainly triterpenoid saponins. In addition, quinoa stalks were reported as a high source of saponins as well (Gil-Ramirez et al. 2018). Where quinoa extract with saponin content of 149.52 ± 2.22 mg/g d.m. was able to reduce surface tension (37.8 ± 0.6 mN/m) (Gόral et al. 2021).

It should be noted that lots number of plants are capable to produce saponins in their biosynthesis pathway during the different stages of plants growth cycles. Nonetheless, limited numbers are oriented as the industrial crops where sustainably supplied for the consumptions in a variety of consumer products. In fact, there are specified regulations for different sorts of product. For cosmetic applications, those that are used are named in term INCI (International Nomenclature of Cosmetic Ingredients) in addition to CAS (Chemical Abstracts Service) and EC (European Community) numbers. Where, the cosmetic substances or ingredients that are regulated to be used in cosmetic products abided with EU regulation can be retrieved from the European Commission database or so called CosIng system.

### Plant saponins and EU cosmetic ingredients cosing

In regard with tremendous plants have been continuously explored upon their potential applications including for cosmetic and personal care products (Bravo et al. 2020). Nonetheless, most of the studies are focus on preparation of the saponins and biological activities. More importance, those that are abided with the list published in the European Commission (CosIng) that are of crucially for cosmetic uses facilitating regulatory of the products are insufficiently demonstrated on the required information, which burden their applications in the sector that continuously increasing on demand for the plants derived materials. This context is therefore aimed to summarize the crops that are registered as plant saponin in the CosIng database. Those that are insufficiently exploited on the information required for cosmetic formulations are therefore encouraged to be examined. In addition, multidirectional cosmetic beneficials of the filled plants saponin would be encouraged to be explored. These industrial crops will be properly knowledge managed for their sustainable utilizations as the bio-based materials promising for cosmetic industrial perspectives.

Saponins listed in the CosIng database are indicated with their INCI name including CAS and EC numbers as well, where the plant sources are indicated (Table 1). In addition, their cosmetic beneficials, i.e., functional and biological activities are addressed as well as those of commercializing available ones (Table 2) together with surface activities and total saponins content (Table 3) of some plant saponins.

**Table 1:** Plant saponins in EU cosmetic ingredients CosIng (https://ec.europa.eu/growth/tools-databases/cosing/)

No.	INCI name	CAS No.	EC No.	Functions	Source
1	SAPONINS	8047-15-2 / 11006-75-0 / 72231-29-9	232-462-6 / - / -	Cleansing, emulsifying,	
2	GINSENOSIDE RD	52705-93-8	258-118-5	Antioxidant	*Acanthopanax senticosus*
3	GINSENOSIDE RE	52286-59-6		Antioxidant, humectant, skin conditioning	
4	GINSENOSIDE RG4	126223-28-7		Antioxidant	
5	GINSENOSIDE RG6	147419-93-0		Antioxidant, skin conditioning and protection	
6	GINSENOSIDE RH4	174721-08-5		Anti-seborrheic, antioxidant, skin conditioning and skin protecting	
7	STARFISH SAPONINS			Skin conditioning	*Asterias amurensis*
8	AVENACOSIDES			Skin conditioning, emollient	*Avena sativa*
9	SAPONINYL ACETOSTEARDIMONIUM CHLORIDE	-	-	Anti-seborrheic, skin and hair conditionings	*Camellia* spp.
10	GINSENOSIDES	-	-	Skin conditioning	*Panax ginseng*
11	GINSENOSIDE RG1			Hair and skin conditionings, humectant	
12	GINSENOSIDE RG2	52286-74-5		Hair and skin conditionings, humectant	
13	GINSENOSIDE RH1	63223-86-9		Hair and skin conditionings, humectant	
14	HYDROLYZED GINSENG SAPONINS	-	-	Skin conditioning	
15	PLATYCODIN D	58479-68-8	-	Skin conditioning	*Platycodon grandiflorus*
16	PULSATILLA SAPONIN	106577-41-7		Skin conditioning	*Pulsatilla chinensis*
17	ZIYU GLYCOSIDE I	-	-	Skin conditioning	*Sanguisorba officinalis*
18	ZIYU GLYCOSIDE II	84787-71-3	284-112-7	Skin conditioning	
19	ZIYU GLYCOSIDE III	84787-71-3	284-112-7	Skin conditioning	
20	PROTODIOSCIN	55056-80-9	-	Skin conditioning	*Tribulus terrestris* and *Trigonella foenumgraecum*

**Table 2:** Commercializing cosmetic substances for plant saponins

Name			CAS No.	Source	Supplier
Saponin	INCI	Trade			
Saponins	Saponins	Bio-saponins^TM^	8047-15-2/ 11006-75-0/ 72231-29-9	sarsaparilla root, wild yam root, quillaja root and yucca root	Bio-botanica
		Tea Saponin (60–90%)	8047-15-2 / 11006-75-0 / 72231-29-9	green tea	Green Source Organics
		Imdermalab^®^ Tea Gem			lmDerma Laboratories
		Tensami^®^ 10 − 06	8047-15-2		Croda
		SILENAGE G	8047-15-2	flowering plant silene colorata	Codif
		Silenys			Barnet
	Camellia Sinensis Seed Extract	SpecPure^®^ BTS Saponins	84650-60-2	Camellia Sinensis seed	
		Slimbuster H		Trichilia Catigua Extract (and) Ptychopetalum Olacoides Bark/Stem Extract (and) Pfaffia Paniculata Root Extract	Chemyunion
	Agave Tequilana Leaf Extract	Actiphyte^TM^ blue agave		Agave Tequilana Leaf Extract	
	Hieracium Pilosella Extract (and) Bellis Perennis (Daisy) Flower Extract	JuvenEye CLR^TM^		triterpene glycosides (saponins)	CLR Chemisches Laboratorium Dr. Kurt Richter GmbH
GINSENOSIDE	PANAX GINSENG ROOT EXTRACT	Morechem Ginseng Extract	84650-12-4	Panax ginseng Root	Morechem
	PANAX GINSENG ROOT EXTRACT	Dermalab Ginseng extract	84650-12-4	Panax ginseng root	Dermalab
	PANAX GINSENG ROOT EXTRACT	Dermalab Red ginseng extract	84650-12-4	Panax ginseng root	Dermalab
	PANAX GINSENG ROOT EXTRACT	TitrExtract^®^ Ginseng	84650-12-4	Panax ginseng root	ID bio
	PANAX GINSENG ROOT EXTRACT	Ginseng Extract-NS	84650-12-4	Panax ginseng root	The Garden of Naturalsolution
	PANAX GINSENG ROOT EXTRACT	Red Ginseng Extract-NS	84650-12-4	Panax ginseng root	The Garden of Naturalsolution
	PANAX GINSENG ROOT	Cosme-Phytami^®^ Ginseng (red)	84650-12-4	Panax ginseng root	Croda
	PANAX GINSENG ROOT EXTRACT	Gerbex^®^ Ginseng	84650-12-4	Panax ginseng root	Shanghai JAKA Biotech
	PANAX QUINQUEFOLIUM ROOT EXTRACT	Panex Ginseng Glycolic Extract^TM^	90045-38-8	PANAX QUINQUEFOLIUM ROOT	Haldin
	PANAX GINSENG ROOT EXTRACT	GINSENG EXTRACT GNBN	50647-08-0 Prosapogenin (Ginseng) GINSENOSIDE RG3	Panax ginseng root	Provital
	HYDROLYZED GINSENG SAPONINS	Pro-Black Extract	GINSENOSIDE RG3	Ginseng	ACT
AVENACOSIDES	AVENA SATIVA KERNEL EXTRACT	Avena Oat Extract WS	84012-26-0	oatstraw	The Herbarie
PLATYCODIN D	Platycodin D	PLATYCODIN D	58479-68-8	Platycodon grandiflorus	Shanghai Tauto Biotech

**Table 3:** Surface activities and total saponins content of plant saponins

Source	Surface activities		Total saponins content	Reference
	CMC	Surface tension		
*Avena sativa*		42.8 ± 0.2 mN/m		Góral et al. 2021
*Camellia* spp.		50.0 mN/m		Chen et al. 2010a
*Chenopodium quinoa*		37.8 ± 0.6 mN/m	149.52 ± 2.22 mg/g d.m.	Góral et al. 2021
*Panex ginseng*		35–40 mN/m		Ralla et al. 2017
*Quaillaja saponaria*	0.14–0.77 g/L			Mitra and Dungan 1997; Wojciechowski et al. 2014
		44.5 mN/m		Benichou et al. 1999
*Sapindus mukorossi*	7.50 × 10^3^ g/cc	35.3 mM/m		Pradhan and Bhattacharyya 2017
*Saponaria officinalis*		49.1 ± 0.3 mN/m	50.92 ± 2.40 mg/g d.m	Góral et al. 2021
*Trigonella foenumgraecum*		33.3 mN/m		Benichou et al. 1999

#### *Acanthopanax senticosus* or *Eleutherococcus senticosus*

This traditional Chinese medicinal herb has been using for neurasthenia, hypertension and ischemic heart disease treatments. Which, triterpenoids saponins were determined as the major constituents of its leaf, and being responsible for the pharmacologically actives confirming the traditionally used of the herb (Xia et al. 2019). Siberian ginseng saponin protected oxidative stress in nerve cell damage examined in an animal model (Pan et al. 2002), where its mechanism on neuroprotective effect was thereafter clarified (Huang et al. 2022).

#### *Avena sativa*

Oat is one of the important industrial crops severing in several products for human consumptions (Singh et al. 2013) includes food, and livestock feed. Oat is a unique plant for triterpenoids and steroidal saponins extractable from different parts of the plant. Saponin extract of oat seeds was demonstrated onto its high reduction of surface tension (42.8 ± 0.2 mN/m) (Gόral et al. 2021). Furthermore, oat triterpenoid saponins, monodesmosidic and bidesmosidic saponins, exhibit antifungal activity by complexing with sterols in fungal membranes that the sugar moiety of saponins is of critical for the anti-microbial activity, where a reduction of sugar molecule substantively reduce the activity (Armah et al. 1999). In addition, oat saponins’ techno-functional properties are tailor-made by means of hydrolysis giving the suitable molecular size viably for different products and textures.

#### *Camellia* spp.

Tea saponins are prepared from tea seed shells with a milky white or pale yellow having the molecular mass of 1200–2800 (Kitagawa et al. 1998). This nonionic saponins posed appointed surface activities, i.e., foaming, emulsifying and wetting (Feng et al. 2015) and capable to reduce water surface tension from 72 mN/m to 50.0 mN/m. Moreover, its antioxidant activity (Chen et al. 2010b) indicates tea saponins’ potency to be used as multifunctional ingredients for cosmetics, and accountable for the self-preserving agents on the basis of inhibitory effects against *Escherichia coli* and *Staphylococcus aureus* (Zhao et al. 2020), and *Bacillus subtilis* the prohibited microbes in cosmetics with MIC and MBC of 31.3 and 62.5, 31.3 and 62.5 and 62.5 and 125 µg/mL (Hu et al. 2012). In addition, tea triterpenoid saponins posed inhibitory effect against hyaluronidase at IC_50_ of 19.3–55.6 µM (Myose et al. 2012) that may applicable for anti-wrinkle cosmetic applications as well.

#### *Panax ginseng*

Saponins are extracted from *Panax* spp. with 70% ethanol at 200 MPa and 60 ºC, where this condition is noted as the optimal condition giving 0.2–0.3% ginsenosides (Becker et al. 2015). Triterpenoid saponins of *P. ginseng*, the amphiphilic ginsenosides can be neutral or acidic natures depend on the desmosidic sugars in its hydrophilic part of the saponins. Which, ginsenosides are bidesmosidic in general. Sugar moieties with carboxylic acid within, the acidic ginsenosides. Ginsenosides were noted onto their high surface activity (Han and Kim 1984; Lee and Lee 1995) of approximately 35–40 mN/m (Ralla et al. 2017) with the determined CMC at 0.009% w/v (Mesgarzadeh et al. 2017). Interestingly, saponin ginsenosides were remarkedly posed anti-inflammatory and anti-cancer activities in addition to their antioxidative capabilities (Kim et al. 2014, 2017).

#### *Platycodon grandiflorus*

This medicinal herb that has been composed in several recipes of traditional Chinese medicine is a promising source of triterpenoid saponins, i.e., platycodin D. Cellular antioxidant activity of the saponins was evidenced (Wang et al. 2004) with the protective effect against hepatotoxicity (Lee et al. 2004). Biological activity against *Candida albicans* was elucidated by a reduction of the microbes in spore phase to mycelium phase with a reduction of adhesive spores onto the contact epithelial cells. In the same time that mRNA levels of IL-8 were suppressed (Zhu et al. 2017). The saponins were reported onto anti-inflammatory effects (Wang et al. 2017) with the negative property against the growth of carcinoma cells (Zhang et al. 2017). Anti-tumor activity of platycodin D was elaborated in several cellular studies, which, the induced apoptosis was by caspase-3-dependent PARP and laminin A (Kim et al. 2005, 2008) promotion of platycodin Dwas In addition, platycodin D posed lipase inhibitory effect, which applicable for utilization in innovative health promotion products in terms of food for lipid metabolic disorder treatments (Han et al. 2002). Moreover, they were shown to reduce blood sugar, serum cholesterol and triglyceride levels in animal models suggesting the saponins’ potency for diabetic treatment as well (Luan et al. 2013).

#### *Pulsatilla chinensis*

Triterpenoid saponins derived from *P. chinensis* are basically constructed with the monosaccharides that are glucose, rhamnose, arabinose and xylose. *Pulsatilla* saponins have diverse therapeutic properties. Anticancer activities of the saponins were exhibited in hepatocellular carcinoma SMMC7721 cells (Xue et al. 2019), pancreatic SW1990 cancer cells (Bolan et al. 2013), breast cancer cell lines (MCF-7 and MDA-MB-231) (Wang et al. 2020) and SW480 colon adenocarcinoma cell line (Zhang et al. 2019). In addition, anti-inflammatory activity was also evidenced in animal models, i.e., rat ex vivo (Hu et al. 2009) and in mice studies (Kang et al. 2019). The saponins additionally functioned as immunomodulators as indicated in mice (Sun et al. 2010). Inhibitory effect of *Pulsatilla* saponins against prohibited microbe in cosmetics, i.e., *Staphylococcus aureus*,* Pseudomonas aeruginosa* and *Escherichia coli*, were reported (Shi et al. 2005).

#### *Tribulus terrestris*

The fruit extract of *T. terrestris* is regulated for skin conditioning in cometics with CAS and EC no. of 90131-68-3 and 290-381-1, respectively (European Commission, *Tribulus terrestris*). Its fruit was reported to be high in saponin content (90.4%), where steroidal saponins constituted with mono- and bidesmosidic sugars were the main homologues. In spite of its surface tension reducing ability, its foamability was poor in regard with its steroidal saponins’ nature (Böttcher and Drusch 2016).

#### *Trigonella foenumgraecum*

The traditional herb Fenugreek is composed with steroid saponins in its seeds. Fenugreek saponins were shown to better in surfactant efficacy than Quillaja (33.3 and 44.5 mN/m). In addition, HLB of fenugreek saponins was determined to be 18 (Benichou et al. 1999).

## Conclusions and future perspectives

Plants are regarded as the reliable source significance for human consumptions, and applied for curtained industries including cosmetic and personal care products. At which, sustainability and circular bioeconomy are of prime focus (Martins and Marto 2023) among the consumers and the researchers. Plants are shaded upon their unique capabilities (Rai et al. 2021, 2023) that adequately serve on the emerging demand for natural- and sustainable-derived products, where the circular bioeconomy is approachable. The new findings and inventions reveal the functional and active tasks of plants appointed for the industrial are exhaustively presented. However, there are some numbers of plants regulated to be used as the cosmetic substances that might obstacles or downregulates cosmetic capability of some plants. In addition, in the point of industrial feasibility, the oriented plants are of significance for sustainability utilization where all parts of the plants can be implied in variety of the consumer products, although in different sectors. Circular bioeconomy is therefore the sustainable approaches for industrial crop productions. This presenting exploration is therefore shed light on the crops management challenge. Where, plants’ saponins should be revisited towards the agricultural practices and biotechnology tools for enhancing plants’ saponins productive yields. In addition, the extracting methods for saponins that are efficient and meeting with the sustainability and bioeconomy themes encouraged to be explored. Furthermore, cosmetic properties and characteristics are of crucially to be concerned in an order to comply with the standard of the industry where innovative and creative products are essential.

## Data Availability

All sources of information are included in this review article.
